# A 3D geometric morphometric analysis of the bovid distal humerus, with special reference to *Rusingoryx atopocranion* (Pleistocene, Eastern Africa)

**DOI:** 10.1111/joa.14062

**Published:** 2024-05-11

**Authors:** Sophia C. Anderson, Kris Kovarovic, W. Andrew Barr

**Affiliations:** ^1^ Department of Archaeology University of York York UK; ^2^ Department of Anthropology Durham University Durham UK; ^3^ Department of Anthropology The George Washington University Washington District of Columbia USA

**Keywords:** bovid, ecomorphology, functional morphology, geometric morphometrics, habitat preference, humerus

## Abstract

The family Bovidae [Mammalia: Artiodactyla] is speciose and has extant representatives on every continent, forming key components of mammal communities. For these reasons, bovids are ideal candidates for studies of ecomorphology. In particular, the morphology of the bovid humerus has been identified as highly related to functional variables such as body mass and habitat. This study investigates the functional morphology of the bovid distal humerus in isolation due to its increased likelihood of preservation in the fossil record, and the resulting opportunity for a better understanding of the ecomorphology of extinct bovids. A landmark scheme of 30 landmarks was used to capture the 3D distal humerus morphology in 111 extant bovid specimens. We find that the distal humerus has identifiable morphologies associated with body mass, habitat preference and tribe affiliation and that some characteristics are shared between high body mass bovids and those living on hard, flat terrain which is likely due to the high stress on the bone in both cases. We directly apply our findings regarding extant bovids to the extinct alcelaphine bovid, *Rusingoryx atopocranion* from the mid to late Pleistocene (>33–45 ka) Lake Victoria region of Kenya. This species is known for some peculiar morphologies including a domed cranium with hollow nasal crests, and having small hooves for a bovid of its size. Another interesting aspect of *Rusingoryx*'s skeletal morphology which has not been addressed is an unusual protrusion on the lateral epicondyle of the distal humerus. Despite considerable individual variation in the *Rusingoryx* specimens, we find evidence to support its historical assignment to the tribe Alcelaphini, and that it likely preferred open grassland habitats, which is consistent with independent reconstructions of the palaeoenvironment. We also provide the most accurate body mass estimate for *Rusingoryx* to date, based on distal humerus centroid size. Overall, we are able to conclude that the distal humerus in extant bovids is highly informative regarding body mass, habitat preference and tribe, and that this can be applied directly to a fossil taxon with promising results.

## Graphical abstract

We use 3D geometric morphometrics to investigate the functional morphology of the distal humerus in extant bovids, finding that habitat preference, body mass and tribe affiliation have identifiable morphological signals. We then use these results to interpret the ecology of the extinct alcelaphine bovid *Rusingoryx atopocranion* and the implications of this for reconstructing the palaeoenvironment of Pleistocene East Africa.

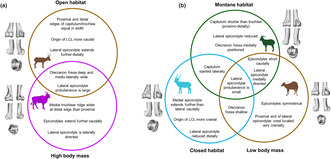

## 1 INTRODUCTION

The family Bovidae [Mammalia: Artiodactyla] is one of the 10 currently recognised families of terrestrial ungulates within the order Artiodactyla (Burgin et al., 2018). Bovids are the most speciose group of extant ungulates, containing approximately 140 species (Grzimek, 2003; Hernández Fernández & Vrba, 2005; Huffman, 2020; Walker, 1999; Wilson & Reeder, 2005). Members of the family are currently found all over the globe in almost every type of terrestrial habitat (Grzimek, 2003). They also range vastly in body mass, from the royal antelope (*Neotragus pygmaeus*) which weighs on average 2.4 kg (Kingdon, 2013), to the water buffalo (*Bubalus bubalis*) which can weigh as much as 1200 kg (Grzimek, 2003). Bovids are a key component of many mammal communities, making up a large proportion of the number of species (and individuals), particularly in the East African savannah where bovid species richness is higher than anywhere else in the world (Grzimek, 2003).

The humerus in bovids is of particular interest, as it plays important roles in locomotion and unguligrade biomechanical efficiency (Hildebrand, 1974). The humerus forms the proximal segment of the forelimb, articulating proximally with the glenoid of the scapula, and distally with the radioulna at the elbow joint. It is both a weight‐bearing structure and an important component of stride production (Biewener, 2003). Its biomechanical role in the proximal forelimb means it has been under evolutionary pressure to adapt to facilitate the unguligrade posture, taking on a greater proportion of the muscle mass so as to reduce weight at the distal limb, as well as providing many of the muscle attachment sites required for the motion of the more distal segments (Clifford, 2010; Hildebrand, 1974). Bovid humerus morphology has been shown to be informative in bovid ecomorphological studies linking humeral morphology to habitat exploitation (Etienne et al., 2021; Kovarovic & Andrews, 2007). Though long bones such as the humerus are regularly found in fossil assemblages, they are often fragmentary due to the susceptibility of the diaphysis to breakage and the epiphyseal ends to carnivore scavenging damage (Bartram & Marean, 1999; Behrensmeyer et al., 2000; Hill & Behrensmeyer, 1984). Even in isolation, the distal portion of the humerus has the potential to be highly informative, particularly due to the large number of muscle attachment sites concentrated in the area which support muscles crucial for movement of the limb below the elbow (almost all extensors and flexors of the carpus and digits). Distal humerus morphology in bovids has also been shown to be a good proxy for body mass using simple linear measurements (Mendoza & Palmqvist, 2006), with the potential for greater insight following detailed and targeted study of the region. For this reason, this study focuses on the functional morphology of the isolated bovid distal humerus.

One extinct bovid for which the distal humerus is well‐represented is *Rusingoryx atopocranion*. This extinct alcelaphine bovid (related to modern wildebeest [*Connochaetes*] and hartebeest [*Alcelaphus*]) is known from the late Pleistocene Wasiriya Beds (~100–36 ka) in the Kenyan portion of Lake Victoria, where it is abundant in the deposits (Jenkins et al., 2017; Kovarovic et al., 2021; O'Brien et al., 2016). *Rusingoryx* is a monotypic genus first described in 1984 from a partial cranium uncovered at the Pleistocene Wasiriya Beds on Rusinga Island in Lake Victoria, Kenya (Pickford & Thomas, 1984), and it is best known for its abnormal hollow nasal crests (O'Brien et al., 2016). It has also been noted to have unusually short phalanges relative to body size, hypothesised to be an adaptation for life on open plains with hard terrain (Kovarovic et al., 2021).

**FIGURE 1:**
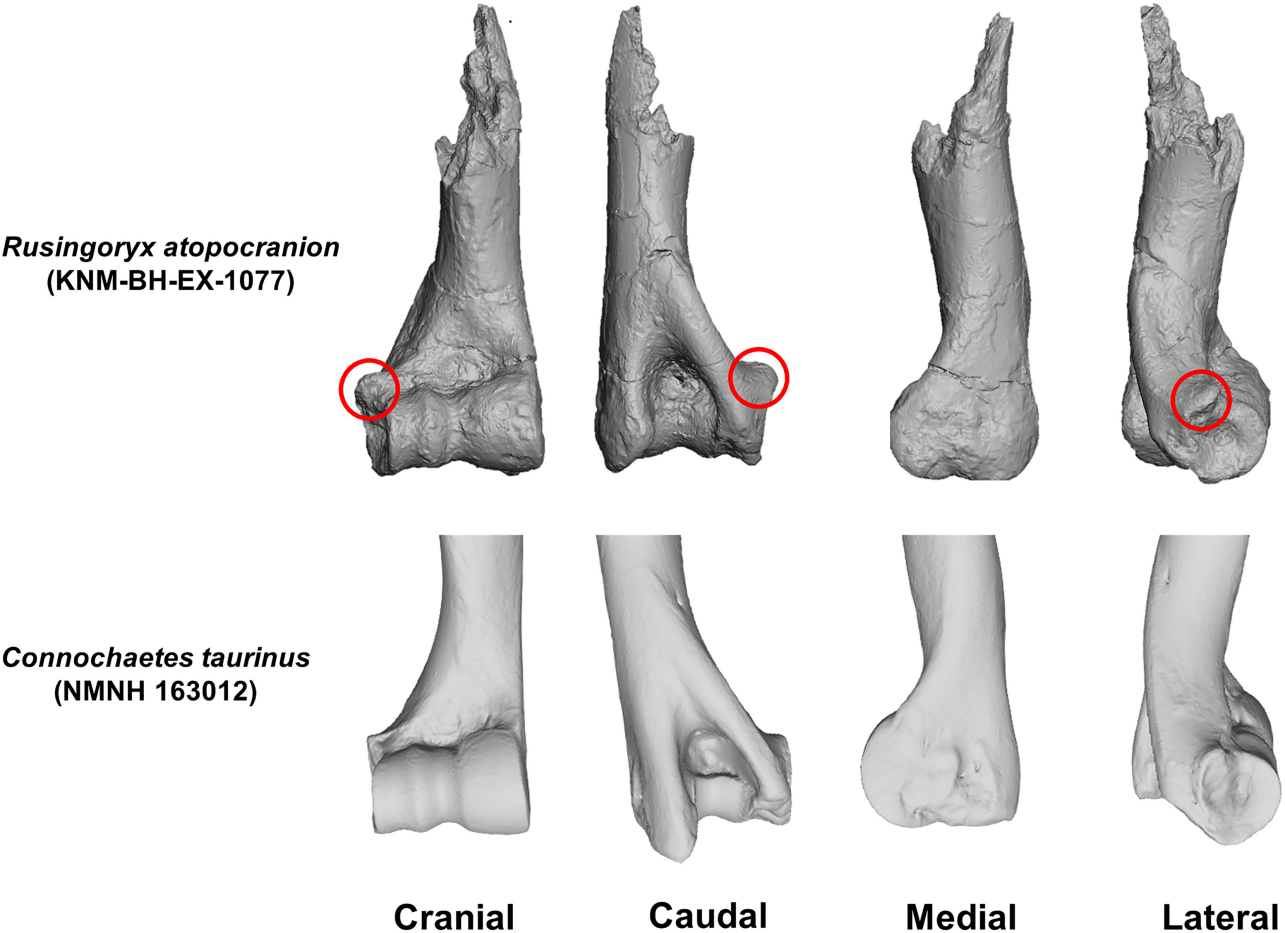
Comparison of the distal humerus in *Rusingoryx atopocranion* and *Connochaetes taurinus*. Showing 3D surface scans of the right distal humerus of *Rusingoryx* (KNM‐BH‐EX‐1077) and *C. taurinus* (NMNH 163012) with the lateral epicondylar protuberance circled in *Rusingoryx*.

The distal humerus of *Rusingoryx* is represented by multiple specimens (held at the National Museums of Kenya, KNM). Importantly for this study, the distal humerus of *Rusingoryx* presents yet another unusual morphology which has yet to be investigated: the lateral epicondyle in multiple specimens exhibits a distinct lateral protuberance which is unusually prominent (Figure 1). The lateral epicondyle provides origin sites for almost all the extensor muscles of the carpus and digits, as well as exhibiting a deep fossa where the lateral collateral ligament originates, which stabilises the elbow joint (Evans & de Lahunta, 2013; Wareing et al., 2011). A small protuberance at the lateral epicondyle can be seen in several bovids to varying extents, being entirely absent in many, but it does not directly provide an attachment site for any muscles, so its functional significance is unclear. Better understanding the functional implications of this morphology will allow us greater insight into *Rusingoryx*'s ecology and, given its abundance in the Pleistocene Rusinga deposits, can provide more information on the palaeoenvironment of the area.

With this in mind, the first aim of this study is to investigate the relationships between distal humerus morphology and (a) habitat preference, (b) body mass and (c) tribe membership. This is investigated using geometric morphometrics (GMM) on a 3D landmark configuration designed to capture areas of anatomical significance as well as overall shape. The data derived from this method were then used to compare morphology within and between body mass groups, habitat preference groups and tribes. Based on previous research (Etienne et al., 2021), we hypothesise that increased body mass will be related to increased humeral robustness and more pronounced muscle attachment sites and that bovids preferring open habitats will also exhibit a robust distal humerus (regardless of body mass) while those preferring forest and montane habitats requiring manoeuvrability will exhibit more gracile and symmetrical distal humeri.

The second aspect of the study is to apply the findings of our ecomorphological investigation to the distal humerus of the extinct bovid *R. atopocranion* in order to better understand the functional significance of its unusual morphology and its implications for our understanding of the habitat in which it lived in the Kenyan portion of Lake Victoria during the Late Pleistocene.

## 2 MATERIALS AND METHODS

### 2.1 Bovid specimens

3D scans of the bovid humerus were obtained for 111 individual specimens representing 54 extant bovid species from 11 tribes (Table 1), many from existing datasets used in previous studies. Additionally, scans of three distal humeri of *Rusingoryx* were acquired. A mean *Rusingoryx* shape was also produced from the Procrustes coordinates of the three real specimens to generate a representative *Rusingoryx* shape. A list of all scanned specimens, their museum location, scanner model and source are given in Supplementary Material 1.

**TABLE 1:** Extant bovid dataset summary with habitat classification and body mass.

Binomial	Tribe	Habitat preference category	Body mass category (kg)	Mean body mass (kg)	No. specimens
*Alceplaphus buselaphus*	Alcelaphini	WBG	90–180	169	2
*Connochaetes gnou*	Alcelaphini	GT	90–180	145	3
*Connochaetes taurinus*	Alcelaphini	GT	180–360	215	3
*Damaliscus lunatus*	Alcelaphini	GT	90–180	117.5	2
*Damaliscus pygargus*	Alcelaphini	GT	45–90	71	2
*Antidorcas marsupialis*	Antilopini	WBG	10–45	29	2
*Antilope cervicapra*	Antilopini	WBG	10–45	37.5	2
*Eudorcas rufifrons*	Antilopini	WBG	10–45	25	1
*Eudorcas thomsonii*	Antilopini	WBG	10–45	19	3
*Gazella dorcas*	Antilopini	GT	10–45	19	3
*Litocranius walleri*	Antilopini	LWB	10–45	40	3
*Nanger dama*	Antilopini	WBG	45–90	57.5	2
*Nanger granti*	Antilopini	WBG	45–90	59.75	3
*Saiga tatarica*	Antilopini	GT	10–45	36	2
*Boselaphus tragocamelus*	Boselaphini	WBG	180–360	205	2
*Tetracerus quadricornis*	Boselaphini	F	10–45	20	2
*Bison bison*	Bovini	GT	>575	679	2
*Bos grunniens*	Bovini	F	360–575	395	2
*Bos javanicus*	Bovini	F	>575	600	2
*Bubalus bubalis*	Bovini	F	>57	700	2
*Bubalus depressicornis*	Bovini	F	180–360	225	2
*Syncerus caffer*	Bovini	LWB	>575	625	3
*Ammotragus lervia*	Caprini	M	45–90	87.5	2
*Capricornis milneedwardsii*	Caprini	M	90–180	112.5	1
*Hemitragus jemlahicus*	Caprini	M	45–90	85	2
*Naemorhedus goral*	Caprini	M	10–45	38.5	1
*Oreamnos americanus*	Caprini	M	90–180	95	2
*Pseudois nayaur*	Caprini	M	45–90	53.5	2
*Rupicapra rupicapra*	Caprini	M	10–45	38	2
*Cephalophus monticola*	Cephalophini	F	1–10	6.25	3
*Cephalophus silvicultor*	Cephalophini	F	45–90	62.5	1
*Sylvicapra grimmia*	Cephalophini	LWB	10–45	18	2
*Addax nasomasculatus*	Hippotragini	GT	90–180	92.5	1
*Hippotragus equinus*	Hippotragini	WBG	180–360	257.5	2
*Hippotragus niger*	Hippotragini	LWB	180–360	205	1
*Oryx dammah*	Hippotragini	WBG	90–180	150.5	2
*Oryx gazella*	Hippotragini	WBG	180–360	227.5	2
*Oryx leucoryx*	Hippotragini	GT	45–90	64.5	2
*Madoqua kirki*	Neotragini	HWB	1–10	4.6	3
*Oreotragus oreotragus*	Neotragini	WBG	10–45	13.5	2
*Ourebia ourebi*	Neotragini	WBG	10–45	12.5	2
*Ovibos moschatus*	Ovibovini	GT	180–360	295	1
*Kobus ellipsiprymnus*	Redunicini	HWB	180–360	217.5	3
*Kobus kob*	Redunicini	LWB	90–180	90.5	3
*Kobus vardonii*	Redunicini	LWB	45–90	69.5	1
*Redunca arundium*	Redunicini	LWB	45‐90	72.5	1
*Redunca fulvorufula*	Redunicini	LWB	10–45	28.5	2
*Redunca redunca*	Redunicini	LWB	45–90	50	3
*Taurotragus derbianus*	Tragelaphini	HWB	>575	675	2
*Taurotragus oryx*	Tragelaphini	HWB	>575	575	2
*Tragelaphus eurycerus*	Tragelaphini	HWB	180–360	293.5	1
*Tragelaphus scriptus*	Tragelaphini	HWB	10–45	39	3
*Tragelaphus spekii*	Tragelaphini	HWB	45–90	87.5	2
*Tragelaphus strepsiceros*	Tragelaphini	HWB	180–360	217.5	2

Abbreviations: F, forest; GT, grassland/treeless; HWB, heavy woodland/bushland; LWB, light woodland/bushland; M, montane; WBG, wooded bushland/grassland.

### 2.2 Habitat preference and body mass categories

A six‐level habitat system was used to classify each extant taxon into a broad habitat category: Grassland/treeless (GT), wooded bushland/grassland (WBG), light woodland/bushland (LWB), heavy woodland/bushland (HWB), Forest (F) and montane (M) (full definitions in the original sources: Barr, 2020; Etienne et al., 2021; Kovarovic et al., 2021). It is important to note that the habitat categories relate to the amount of vegetation cover, not the type of cover itself.

A seven‐level body mass classification system was used following Kovarovic and Andrews (2007): 1–10, 10–45, 45–90, 90–180, 180–360, 360–575 and >575 kg. Body mass values were obtained from Kingdon (2013) and Etienne et al. (2021). Category assignments for each species can be found in Table 1.

### 2.3 3D landmarking

**FIGURE 2:**
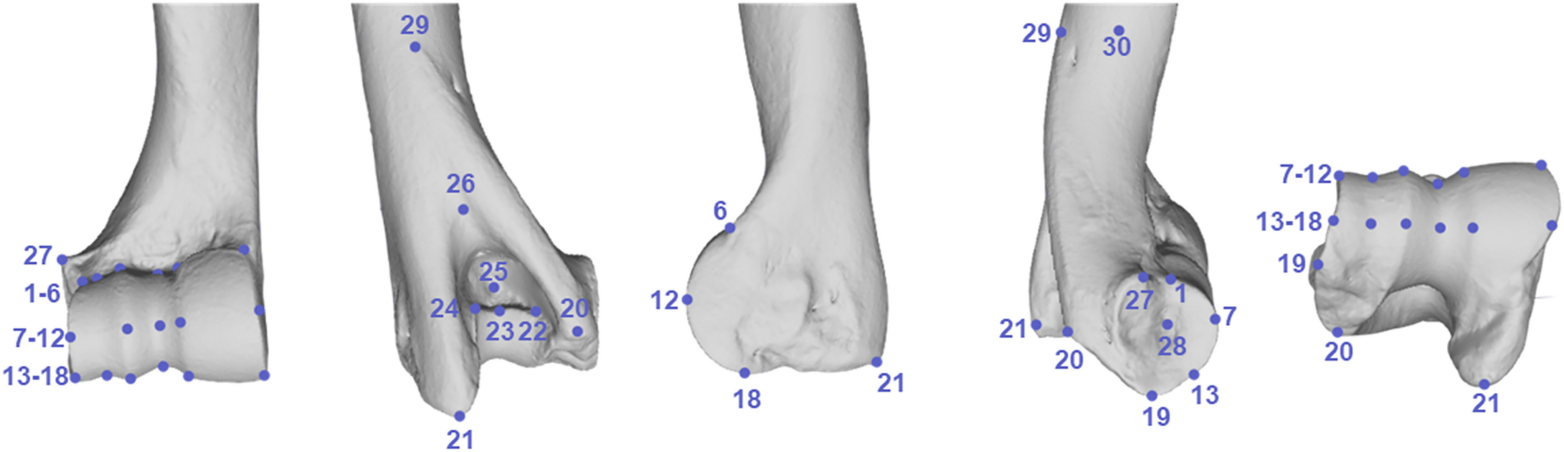
3D landmarks used in this study. Distal humerus of *Connochaetes taurinus* (NMNH 163012) showing the placements of the 30 landmarks used in this study in cranial, caudal, medial, lateral and distal views.

A landmark configuration of 30 true landmarks (Figure 2; Table 2) was used for this study. The choice of landmarks was based on anatomical significance, as well as accurate overall shape representation and repeatability. This does not include interobserver repeatability, which was not measured in this study, as all landmarking was carried out by a single author (SCA). Landmarking was performed in Avizo v7.1.0 (Konrad‐Zuse‐Zentrum Berlin, 2012).

**TABLE 2:** 3D landmark definitions used in this study.

Landmark number	Description
1	Point where proximal end of the lateral border of the capitulum meets the diaphysis
2	Point where proximal end of the groove of the capitulum meets the diaphysis
3	Point where proximal end of the lateral trochlear ridge meets the diaphysis
4	Point where proximal end of the groove of the trochlea meets the diaphysis
5	Point where proximo‐lateral corner of the medial trochlear ridge meets the diaphysis
6	Point where proximo‐medial corner of the medial trochlear ridge meets the diaphysis
7	Point at 50% height of the lateral border of the capitulum in cranial view
8	Point at 50% height of the groove of the capitulum in cranial view
9	Point at 50% height of the lateral trochlear ridge in cranial view
10	Point at 50% height of the groove of the trochlea in cranial view
11	Point at 50% height of the lateral edge of the medial trochlear ridge in cranial view
12	Point at 50% height of the medial edge of the medial trochlear ridge in cranial view
13	Point at 50% depth of lateral border of the capitulum in distal view
14	Point at 50% depth of the groove of the capitulum in distal view
15	Point at 50% depth of the lateral trochlear ridge in distal view
16	Point at 50% depth of the groove of the trochlea in distal view
17	Point at 50% depth of the lateral edge of the medial trochlear ridge in distal view
18	Point at 50% depth of the medial edge of the medial trochlear ridge in distal view
19	Most distal extension of the lateral epicondyle
20	Most caudo‐distal extension of the lateral epicondyle
21	Most caudo‐distal extension of the medial epicondyle
22	Lateral interior corner of olecranon fossa
23	Point on the distal border of the olecranon fossa equidistant between landmarks 22 and 24
24	Medial interior corner of olecranon fossa
25	Cranio‐caudally deepest point of olecranon fossa
26	Most proximal point of olecranon fossa
27	Most lateral extent of protuberance on lateral epicondyle
28	Deepest point of fossa for lateral collateral ligament
29	Most proximal point of lateral epicondylar crest
30	Point at 50% cranio‐caudal width of diaphysis in line with landmark 29 in medial view

### 2.4 Statistical analysis

The landmark coordinate data were imported into MorphoJ ver. 1.07a (Klingenberg, 2011) where a Procrustes superimposition was carried out to standardise for variations in scaling, rotation and translation within the 3D space (Gower, 1975; Zelditch et al., 2012). The resulting Procrustes coordinates are thus analysed for variation in shape only.

The following analyses were performed on the Procrustes coordinates acquired in MorphoJ. Principal components analysis, canonical variate analysis and linear regressions were performed in R v4.0.3 (The R Foundation for Statistical Computing, 2020). Where *p*‐values are calculated, significance is established at *p* ≥ 0.05 and exact values are not provided where *p* < 0.001.

Principal component analysis (PCA): This multivariate analysis reduces the dimensionality of the Procrustes coordinate data matrix and presents information on which components are responsible for the greatest variation in the dataset. Visualisation of the PCA axes indicates which individuals are most similar and which are most distinct, as well as which aspects of morphology are driving this.

Canonical variate analysis (CVA): This multivariate analysis uses categorical variable group membership to establish shape components which unify members of a group, and distinguish the groups from one another. Visualisation of the CVA axes indicates the relative ability of the analysis to distinguish groups and the aspects of morphology which drive the differentiation. Cross‐validation was performed as part of the CVA to compensate for over‐fitting (Barr & Scott, 2014; Kovarovic et al., 2011), and prediction accuracies are presented following this.

Regression: Linear regression is used to investigate the relationship between two linear variables, while multivariate regression is used to investigate the relationship between a multivariate variable and either another multivariate variable, or a linear variable.

Phylogenetic generalised least squares (PGLS): This analysis aims to evaluate and mitigate the effects of phylogeny on potential form‐function relationships. It is used here to evaluate the significance of PCA and CVA results when phylogenetic relationships are considered. PGLS was performed on multivariate regressions of canonical variate or principal component versus categorical variable (e.g. PC1 ~ Habitat preference). All results are presented following PGLS, thus values of significance reflect significance following PGLS. The phylogeny used for PGLS analysis was taken from VertLife.org. PGLS was performed in R.

## 3 RESULTS

### 3.1 Principal components analysis

**FIGURE 3:**
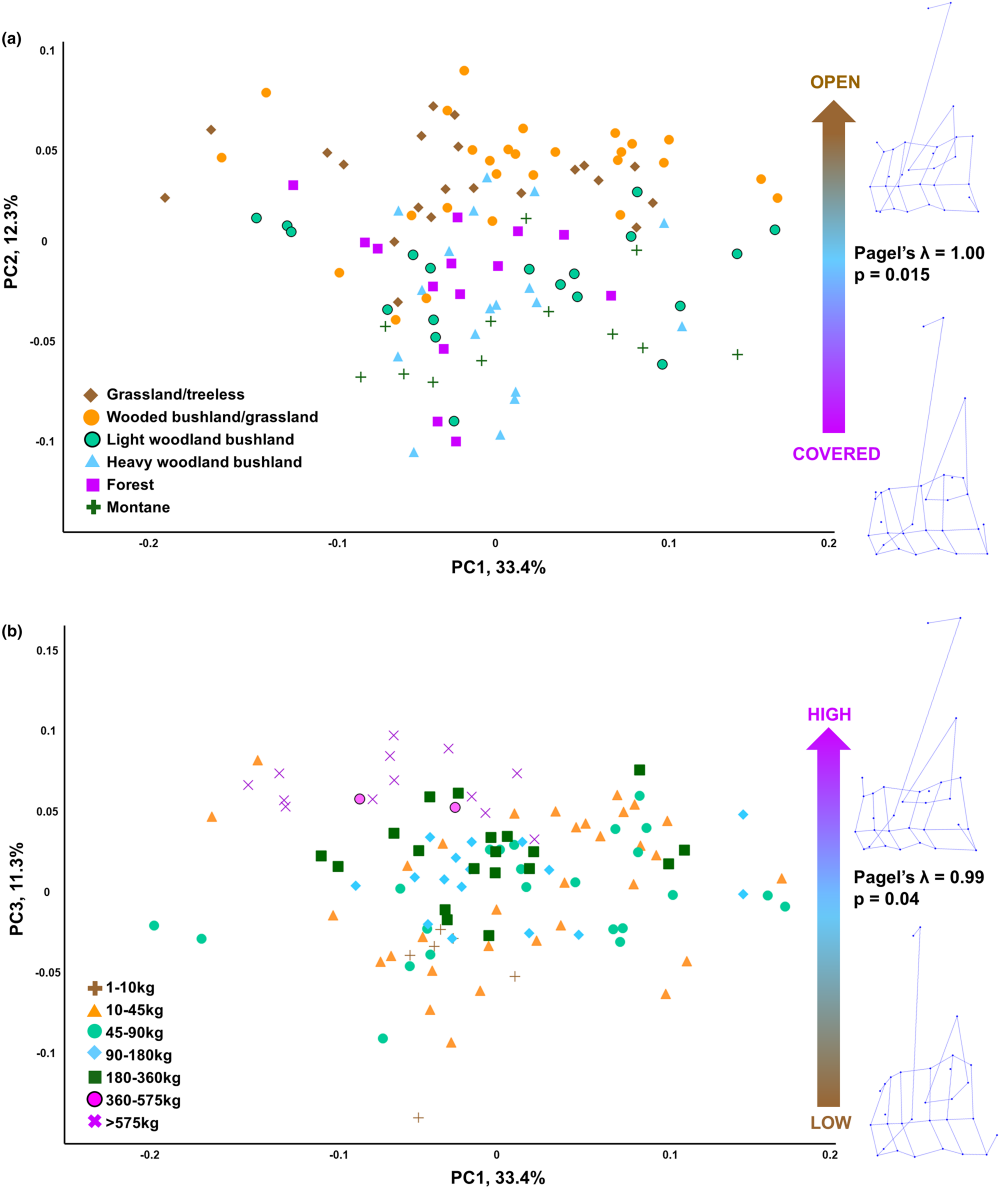
Visualisation of principal component analysis (PCA) on extant bovids by (a) habitat preference and (b) body mass. Plots showing results from a PCA performed on the 3D Procrustes coordinates representing distal humerus shape. Category assignments for both habitat preference and body mass are represented by colour‐coding as well as point shape. (a) PC1 and PC2 axes. The arrow indicates the trend on PC2 for increased open habitat preference, with value for Pagel's lambda (*λ*) and *p*‐value for the relationship between PC2 and habitat preference. (b) PC1 and PC3 axes. The arrow indicates the trend on PC3 for increasing body mass, with value for Pagel's lambda (*λ*) and *p*‐value for the relationship between PC3 and body mass category. Wireframes (acquired from MorphoJ) indicate shape change along the PC2 and PC3 axes.

In a PCA of the 3D Procrustes coordinates, there is no visual separation in any of the first four axes (the first three axes of which are shown in Figure 3) of any habitat preference or body mass categories (variation accounted for by each PC [Table 3]: PC1 = 33.4%; PC2 = 12.3%; PC3 = 11.3%; PC4 = 5.1%). Variation on PC1 appears to be driven by phylogenetic affiliation (multivariate regression of PC1 against habitat preference, Pagel's lambda = 0.92; PC1 against body mass category, Pagel's lambda = 0.91) and is not significantly correlated with habitat preference or body mass (*p* = 0.46 and 0.71 respectively). PC2 and PC4 are significantly correlated with habitat preference (*p*‐values: PC2 = 0.015; PC4 = 0.024), and PC3 is correlated with body mass (*p* = 0.040). However, all of the first four PCs show a strong phylogenetic influence on variation in the dataset (Pagel's lambda: PC2 = 1.00; PC3 = 0.99; PC4 = 0.62).

**TABLE 3:** PGLS results summary of PCs against habitat preference and body mass.

	Percentage variance	PGLS habitat preference		PGLS body mass	
		Lambda (*λ*)	*p*‐value	Lambda (*λ*)	*p*‐value
PC1	33.45	0.92	0.46	0.91	0.71
PC2	12.30	1.00	0.02	1.00	0.04
PC3	11.26	0.99	0.79	0.94	<0.001
PC4	5.09	0.62	0.02	0.88	0.55

By inference from PC loadings and warp visualisations, shape change on PC2 (pertaining to increasing preference for open habitats) is primarily in the form of increasing size of the lateral epicondylar protuberance, deeper and medio‐laterally wider olecranon fossa, and more symmetrical lateral and medial epicondyles (with respect to size). Shape change on PC3 (pertaining to increased body mass) is also primarily related to increasing size of the lateral epicondylar protuberance and deeper and medio‐laterally wider olecranon fossa, and, in addition, increasing caudal extent of the medial epicondyle relative to the lateral epicondyle.

### 3.2 Habitat preference

**FIGURE 4:**
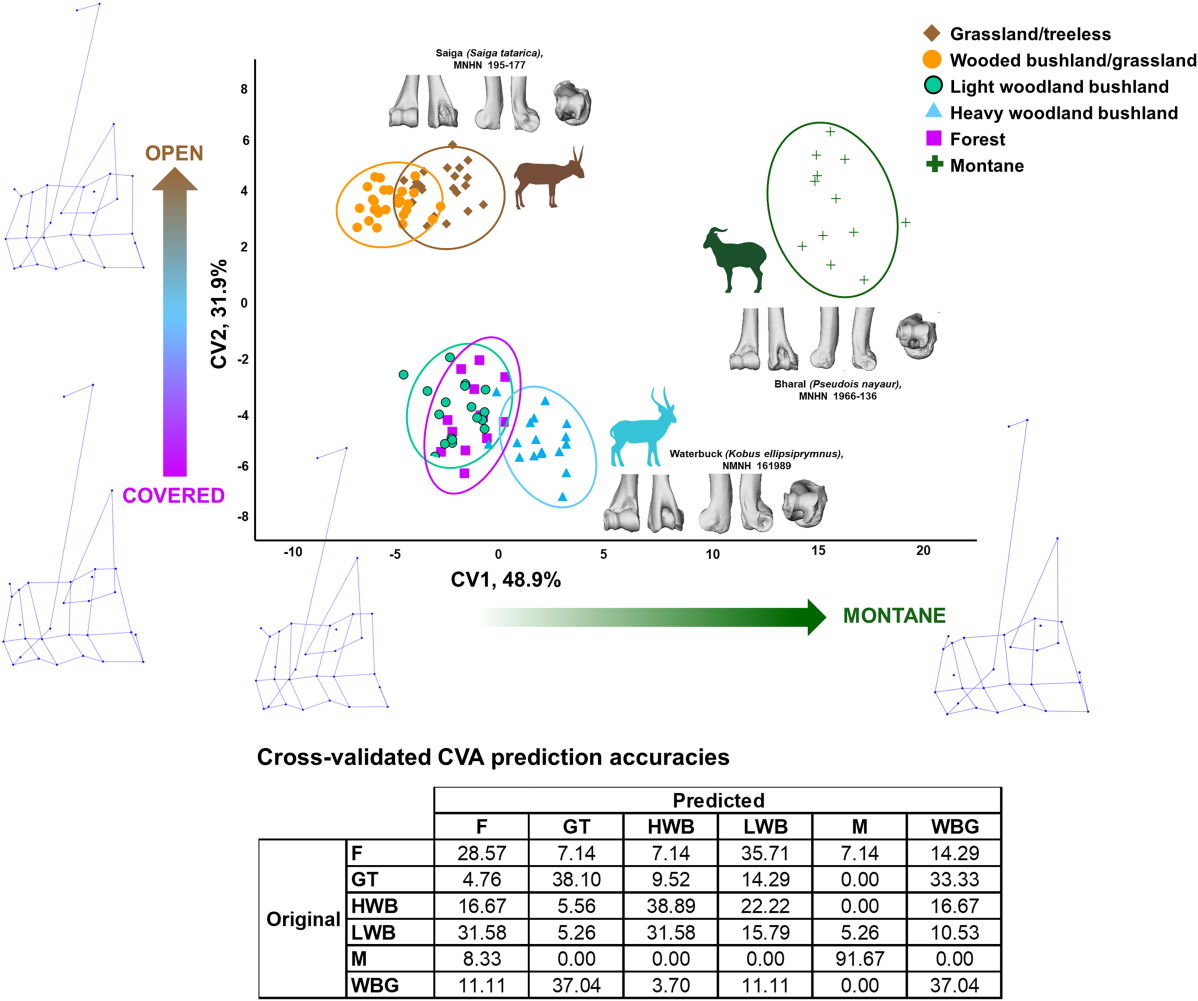
Visualisation of canonical variate analysis (CVA) by habitat preference in extant bovids. Plot showing the first two CVs of a CVA performed on the 3D Procrustes coordinates representing distal humerus shape, with habitat preference as the categorical variable. Ellipses indicate 90% confidence intervals. The table provides percentage prediction accuracies, as compared to original group membership, following cross‐validation. Representative examples of the distal humerus for main clusters are provided in cranial, caudal, medial, lateral and distal views. Bovid silhouette sources: *Saiga tatarica* edited from Kenneth W. Fink photograph; *Kobus ellipsiprymnus* from PhyloPic; *Pseudois nayaur* edited from www.dimensions.com. Wireframes (acquired from MorphoJ) indicate shape change along the axes. F, forest; GT, grassland/treeless; HWB, heavy woodland/bushland; LWB, light woodland/bushland; M, montane; WBG, wooded bushland/grassland.

A CVA by habitat preference reveals a strong potential for distal humerus morphology to differentiate habitat preference groups, but not with high visual differentiation of some morphotypes on the first two axes (CV1 accounts for 48.9% of variation in the data, and CV2 accounts for 31.9%). The first four CVs are significantly related to habitat preference in a PGLS (*p*‐values all <0.001) (Table 4). The montane group separates clearly from all other groups on the CV1 axis (Figure 4), while the more open‐living bovids (categories: grassland/treeless and wooded bushland/grassland) are separated from those preferring some cover (categories: light woodland/bushland, heavy woodland/bushland, and forest) on the CV2 axis. This CVA has a cross‐validated prediction accuracy of 38.7%. While this prediction accuracy is relatively modest, we use more categories than commonly used in ecomorphology studies (typically 3 or 4), which reduces the classification accuracy. Importantly, we can see from the table in Figure 4, that the highest misclassification rates result in misclassification into the next most similar habitat group (e.g. GT is most commonly misclassified as WBG). The only group for which this is not true is the forest group, for which classification may be less accurate.

**TABLE 4:** PGLS results summary of CVs against habitat preference and body mass.

	PGLS habitat preference			PGLS body mass		
	% variance	Lambda (*λ*)	*p*‐value	% variance	Lambda (*λ*)	*p*‐value
CV1	44.88	0.09	<0.001	58.45	0.00	<0.001
CV2	31.88	0.26	<0.001	21.87	0.00	<0.001
CV3	16.75	0.18	<0.001	11.92	0.00	<0.001
CV4	5.03	0.00	<0.001	5.98	0.00	<0.001

The montane bovids are the most morphologically distinct of the groups. They are distinguished primarily by the relative heights (proximo‐distally) of the capitulum on the lateral side and the trochlea on the medial side, with the capitulum being noticeably shorter. The capitulum and, by extension, the lateral epicondyle, are relatively small for the size of the distal humerus. The epicondyles do not extend as far caudally in the montane bovids as they do in other bovids, and they are of equal length cranio‐caudally. The lateral epicondylar protuberance is barely present or entirely absent.

The CV2 axis separates the most open‐living species from those preferring some degree of cover, and these two groups can be differentiated morphologically in a number of ways. In the bovids preferring some level of cover, the proximal edge of the capitulum/trochlea is positioned more laterally than the distal edge, such that the capitulum and trochlea are slanted. Particularly, the proximo‐medial corner of the medial trochlear ridge is highly medially positioned, whereas in the open‐living bovids it falls proximo‐distally in line with the disto‐medial corner. The medial epicondyle extends further than the lateral epicondyle in the caudal direction. In the more open‐living bovids, the proximal and distal edges of the capitulum/trochlea are aligned and symmetrical in height on the medial and lateral edges, and the medial and lateral epicondyles extend to an equal degree caudally. The origin of the lateral collateral ligament is more caudal than bovids preferring cover, and the olecranon fossa is deep and medio‐laterally wide. The lateral epicondylar protuberance is larger in open‐living bovids than those preferring cover.

### 3.3 Body mass

**FIGURE 5:**
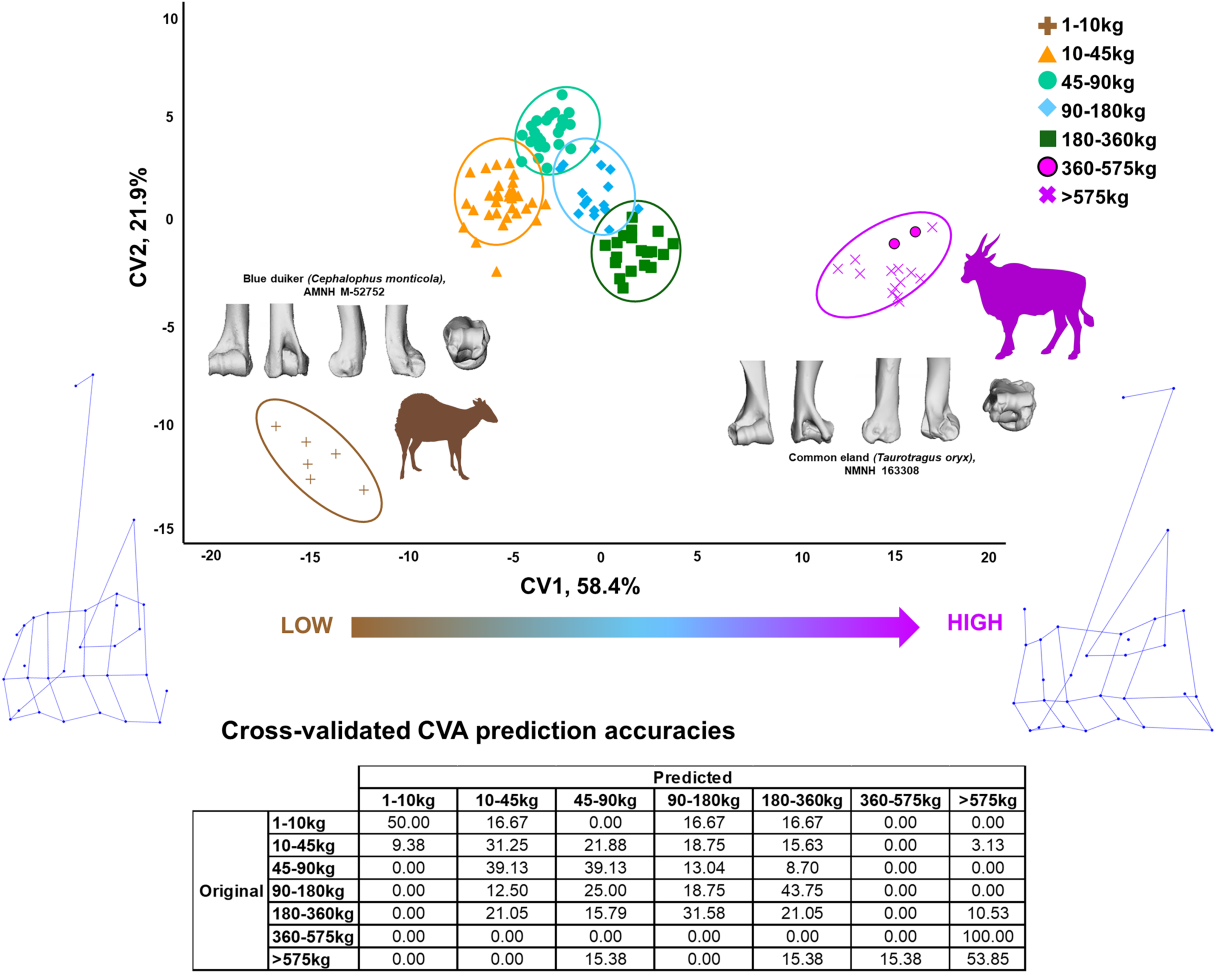
Visualisation of canonical variate analysis (CVA) by body mass category in extant bovids. Plot showing the first two CVs of a CVA performed on the 3D Procrustes coordinates representing distal humerus shape, with body mass as the categorical variable. Ellipses indicate 90% confidence intervals. The table provides percentage prediction accuracies, as compared to original group membership, following cross‐validation. Representative examples of the distal humerus for the smallest and largest masses are provided in cranial, caudal, medial, lateral and distal views. Bovid silhouette sources: PhyloPic. Wireframes (acquired from MorphoJ) indicate shape change along the CV1 axis.

The CVA by body mass reveals a strong potential for distal humerus morphology to differentiate body mass groups, but not with high visual differentiation of some morphotypes on the first two axes (CV1 accounts for 58.4% of variation in the data, and CV2 accounts for 21.9%). The first four CVs are significantly related to body mass in a PGLS (*p*‐value <0.001 respectively) (Table 4). The smallest mass category (1–10 kg) separates entirely from the other categories on the CV1 axis (Figure 5), while categories ranging from 10 to 360 kg cluster closely together and successive mass categories show considerable overlap with one another, then finally the highest two categories (360–575 and >575 kg) cluster closely with some overlap. Broadly, mass increases along the CV1 axis. This CVA has a cross‐validated prediction accuracy of 32.4%. As described in the habitat analysis, this prediction accuracy is modest but reflects the high number of body mass categories used here. As was the case in habitat preference, where misclassifications occur in body mass, it is most likely that an individual will be misclassified into the next most similar body mass category (e.g. 1–10 kg is most commonly misclassified as 10–45 kg).

CV1 fully separates out the lowest body mass bovids (1–10 kg) at the low end and the highest body mass bovids (360+ kg) at the highest end, with all other body mass groups clustering centrally. In the heaviest bovids, the medial trochlear ridge is taller proximo‐distally than the capitulum, which itself is considerably shorter than the lateral epicondyle. The articular portion of the distal humerus is notably wider medio‐laterally than the diaphysis, to a greater relative extent than in the lighter bovids, extending outward laterally. The lateral epicondyle extends caudally considerably further than the medial epicondyle and, the lateral epicondylar protuberance is prominent. Lastly, the olecranon fossa is deep and medio‐laterally wide.

In the lightest bovids, the medial trochlear ridge and the capitulum are much closer in proximo‐distal height. The lateral and medial epicondyles extend caudally to approximately equal degrees. The lateral epicondylar protuberance is reduced or absent. Lastly, the olecranon fossa is shallower and medio‐laterally thin than in the heavier bovids.

**FIGURE 6:**
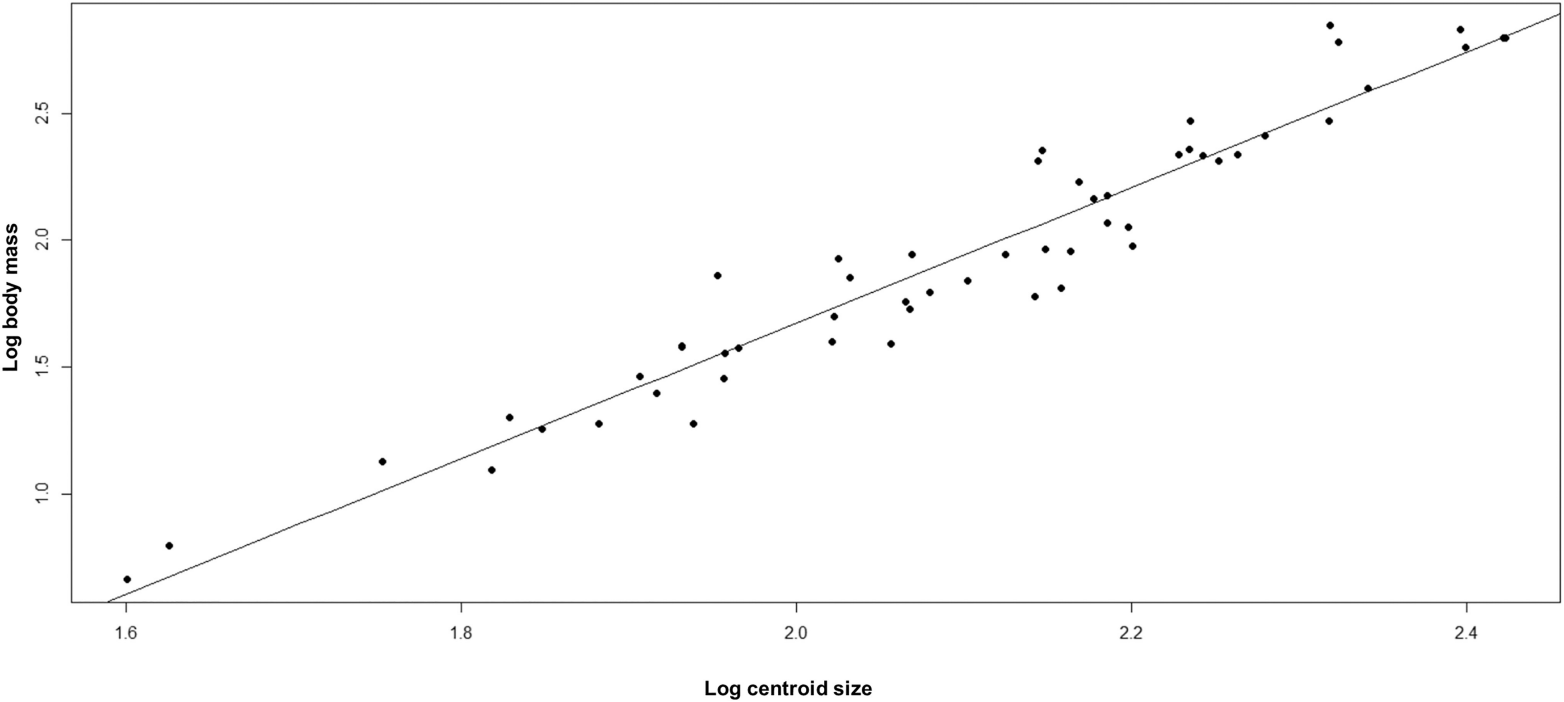
Linear relationship between log body mass and log centroid size in extant bovids. Scatter plot of log body mass against log centroid size. Log(body mass) = (2.65177) × Log(centroid size) − 3.6334, *p* < 0.001, adjusted *r* ^2^ = 0.929, *n* = 111.

However, of arguably greater interest for predictive purposes, we find that there is a significant relationship between log body mass and log centroid size of the distal humerus in extant bovids as follows (logs are to the base 10): log(body mass) = 2.6687 × log(centroid size) − 3.6633 (Figure 6) (*n* = 111, adjusted *r* ^2^ = 0.93, *p* < 0.001).

### 3.4 Tribe affiliation

**FIGURE 7:**
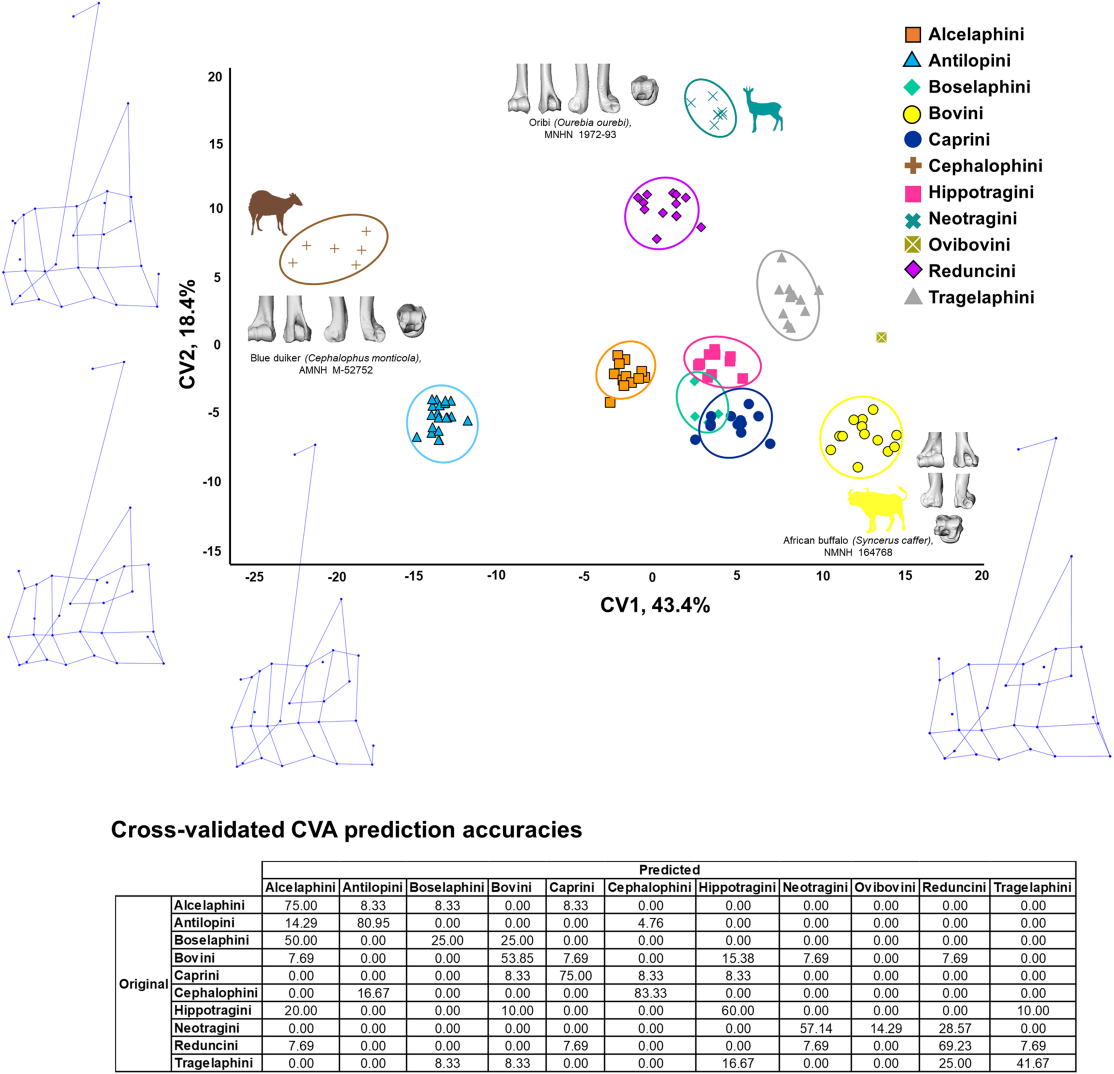
Visualisation of canonical variate analysis (CVA) by tribe in extant bovids. Plot showing the first two CVs of a CVA performed on the 3D Procrustes coordinates representing distal humerus shape, with tribe as the categorical variable. Ellipses indicate 90% confidence intervals. The table provides percentage prediction accuracies, as compared to original group membership. Representative examples of the distal humerus for extremes of the axis are provided in cranial, caudal, medial, lateral and distal views. Bovid silhouette sources: *Cephalophus monticola* from PhyloPic; *Syncerus caffer* from PhyloPic; *Ourebia ourebi* edited from photograph by Jan Dekker. Wireframes (acquired from MorphoJ) indicate shape change along the axes. N.B. The tribe Ovibovini is omitted from this table as the dataset contains one specimen and, as such, no value can be calculated here.

A CVA by tribe (Figure 7) reveals a strong potential for distal humerus morphology to differentiate tribes, with reasonably good visual separation of the morphotypes on the first two axes (CV1 accounts for 43.4% of variation in the data, and CV2 accounts for 18.4%). The CV1 axis separates Cephalophini from Bovini, and the CV2 axis separates the Neotragini and Reduncini from the other bovids. This CVA has a cross‐validated prediction accuracy of 65.5%. This is despite the high number of groups included here, which can be more likely to result in misclassification.

On CV1, the medio‐lateral width of the capitulum/trochlea increases, the lateral epicondylar crest becomes shorter (proximo‐distally), the medial epicondyle extends further caudally while the lateral epicondyle does not, and the olecranon fossa becomes deeper. On CV2, the distal humerus becomes increasingly gracile and relatively elongated proximo‐distally, and the olecranon fossa becomes shallower with its deepest point increasingly medially positioned.

It should be noted that tribe is significantly related to body mass (*p* < 0.001), and body mass has been shown to be significantly related to habitat preference (Supplementary Material 2). Only CV3 of the tribe CVA is significantly related to body mass (*p* < 0.001), but all of the first four CVs are significantly related to habitat (*p* < 0.001). This, in combination with the strong phylogenetic influence on the PCA results, reveals a complex and nuanced interplay of phylogeny and function affecting this morphology.

### 3.5 Rusingoryx atopocranion

**FIGURE 8:**
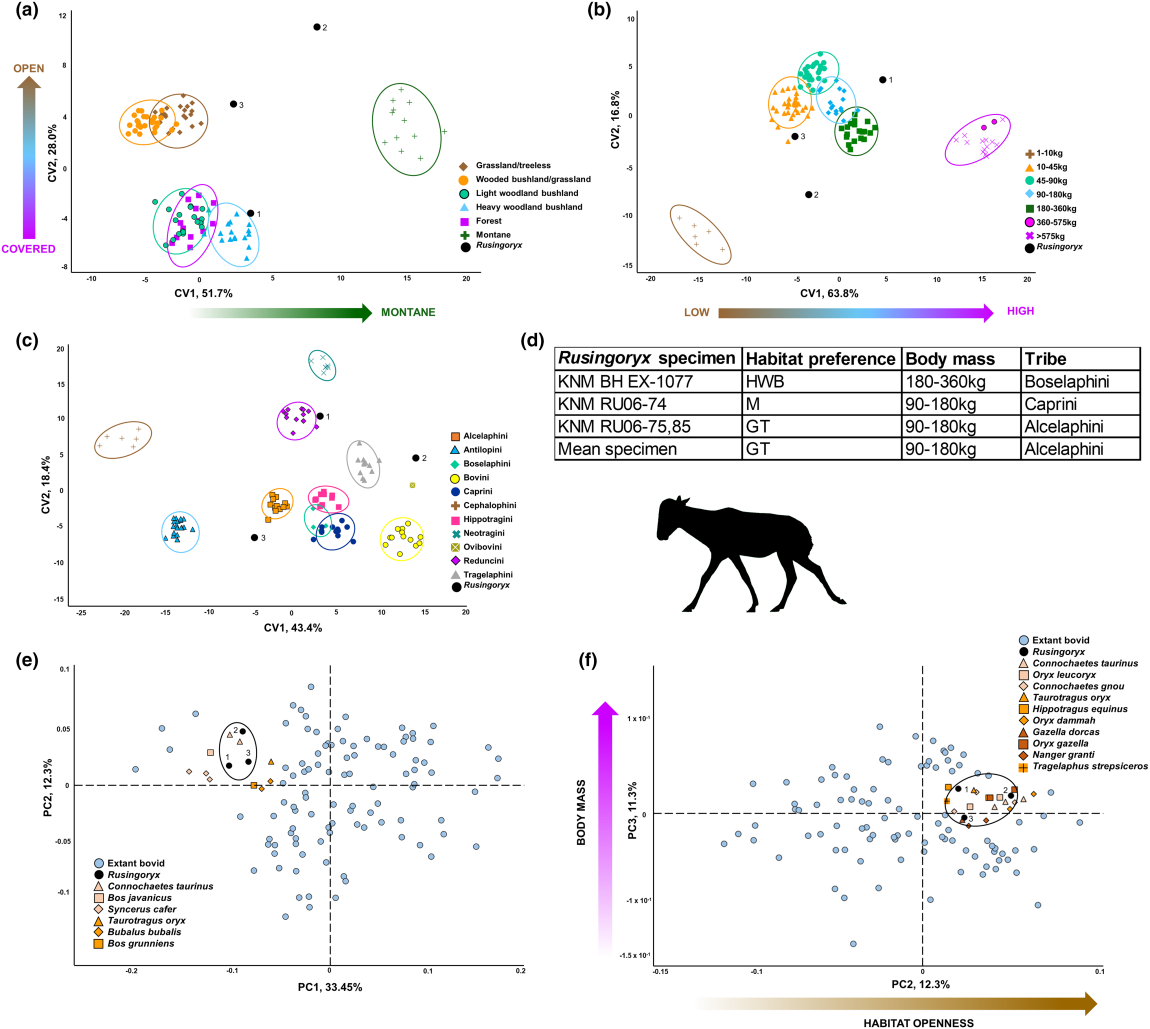
Visualisation of multivariate analysis results including *Rusingoryx*. Plots showing CVA and PCA visualisation using the 3D Procrustes coordinates representing distal humerus shape in extant bovids as well as *Rusingoryx*. *Rusingoryx* specimens are represented by black points and are numbered according to which specimens they represent: 1 = KNM BH EX‐1077; 2 = KNM RU06‐74; 3 = KNM RU06‐75,85. (a) Plot showing the first two CVs of a CVA by habitat preference. Arrows indicate habitat preference trends on both CVs. (b) Plot showing the first two CVs of a CVA by body mass category. Arrow indicates the trend for decreasing body mass on CV1. (c) Plot showing the first two CVs of a CVA by tribe. (d) Table showing classifications assigned by the CVAs to each of the three *Rusingoryx* specimens, as well as the mean specimen shape (N.B. the mean shape is not included in any of the CVA or PCA visualisation). (e) Plot showing PC1 and PC2 of the PCA, visually divided into four quadrants. Individuals close to the *Rusingoryx* specimens in the morphospace are identified. (f) Plot showing PC2 and PC3 of the PCA, visually divided into four quadrants. Individuals close to the *Rusingoryx* specimens in the morphospace are identified. Arrows indicate a trend for increasing habitat openness on PC2 and increasing body mass on PC3. *Rusingoryx atopocranion* silhouette by S. C. Anderson. CVA, canonical variate analysis; GT, grassland/treeless; HWB, heavy woodland/bushland; M, montane; PCA, principal components analysis.

In a visualisation of the habitat CVA (Figure 8a), it can be seen that there is considerable variation between the three *Rusingoryx* specimens, predominantly on the CV2 axis. This axis relates to the shape of the lateral capitulum/trochlea. Interestingly, all three specimens of *Rusingoryx* fall higher on CV1 than the extant bovids they are in line with on CV2. This indicates that *Rusingoryx* is not as morphologically distinct from montane bovids as extant non‐montane bovids are and that it is not clearly aligned with any of the extant groups. The three specimens are predicted by the CVA (38.7% accuracy) to belong to the habitat preference groups HWB, M and GT respectively.

In a visualisation of the body mass CVA (Figure 8b), *Rusingoryx* falls within the morphospace of the majority of the mass categories (masses of 10–360 kg all being represented here). As is the case in the habitat preference CVA, morphological variation between *Rusingoryx* specimens is evident. The three specimens are predicted by this CVA (32.4% accuracy) to belong to the mass categories 180–360, 90–180, and 90–180 kg respectively.

In visualisation of the tribe CVA (Figure 8c), there is, again, considerable variation in the position of the three *Rusingoryx* specimens, though they are clearly differentiated from Cephalophini and Antilopini on CV1, and from Neotragini on CV2. The three specimens are predicted by this CVA (65.5% accuracy) to belong to the tribes Boselaphini, Caprini and Alcelaphini respectively.

A visualisation of PC1–PC2 (Figure 8e) provides limited information, though we can observe the species most morphologically similar to *Rusingoryx* (such as blue wildebeest, *Connochaetes taurinus* and banteng, *Bos javanicus*). As identified above, PC2 is significantly related to habitat preference, and PC3 is significantly related to body mass category. Thus, *Rusingoryx*'s place in the morphospace is informative. In a visualisation of PC2–PC3 (Figure 8f), it can be seen that the *Rusingoryx* specimens load high on PC2 and PC3, mostly lying in the top right quadrant of the plot. This places *Rusingoryx* alongside the most open‐living (GT and WBG), highest body mass bovids (>180 kg) such as blue wildebeest (*C. taurinus*), roan antelope (*Hippotragus equinus*) and the common eland (*Taurotragus oryx*).

When the CVAs for extant bovids are used to predict the group assignment of *Rusingoryx* based on the three specimens (Figure 8d), the analysis fails to consistently predict each of the three specimens to the same category likely due to variable taphonomic damage to the specimens. However, taking a mean of the Procrustes coordinates to create a ‘mean *Rusingoryx*’ (Figure 8d), results in this mean specimen being predicted to the tribe Alcelaphini, to the grassland/treeless habitat (GT) group, and to fall within the mass category 90–180 kg.

We can acquire a much more accurate estimate of *Rusingoryx*'s body mass using the relationship between centroid size and body mass established above. Two of the *Rusingoryx* specimens (BH EX‐1077 and RU06‐75,85) have the same log centroid size of 2.16, while the final specimen (RU06‐74) has a slightly larger log centroid size of 2.18. Using these figures and the relationship between log body mass and log centroid size identified in extant bovids above, we get a body mass estimate for *Rusingoryx* of 125.8–141.25 kg.

## 4 DISCUSSION

The results obtained in this study reflect the anticipated ecologically related morphological adaptability of the distal humerus in bovids, demonstrating that aspects of the morphology are shared by animals with the same habitat preferences or approximate body mass. However, we also find evidence of a strong phylogenetic component to distal humerus morphology, and this must be taken into account before the effect of other factors can be interpreted. It is evident that this complex structure is influenced by multiple factors—those presented here and, undoubtedly, other factors not considered in this study. From the significance of the relationship between shape and both habitat preference and body mass following PGLS analysis, we can infer a strong form‐function relationship in the distal humerus of extant bovids observed across these analyses, even accounting for a significant interaction between habitat preference and body mass in extant bovids.

We find that bovids whose habitat requires movement in open environments (the open habitat preferences) are clearly differentiated from those whose habitat requires agility and manoeuvrability (the covered habitat preferences) due to the different functional demands presented by these habitats. However, the montane bovids appear to have the most morphologically distinct distal humerus of the extant bovids in this study, reflecting the functional demands of vertical manoeuvrability on terrain that is not flat.

We also find that the smallest (1–10 kg) and largest bovids (>575 kg) are the most clearly differentiated, with the intermediate categories (10–360 kg) being less distinct from one another. This likely reflects the increased specialisation required to deal with functional demands at either end of the body mass range, that is, the largest bovids must be highly specialised for weight‐bearing to resist the forces which high body mass exerts on the skeleton, while the smallest bovids must be adapted to have a skeleton able to resist normal forces associated with locomotion, while minimising size (Biewener, 1989).

**FIGURE 9:**
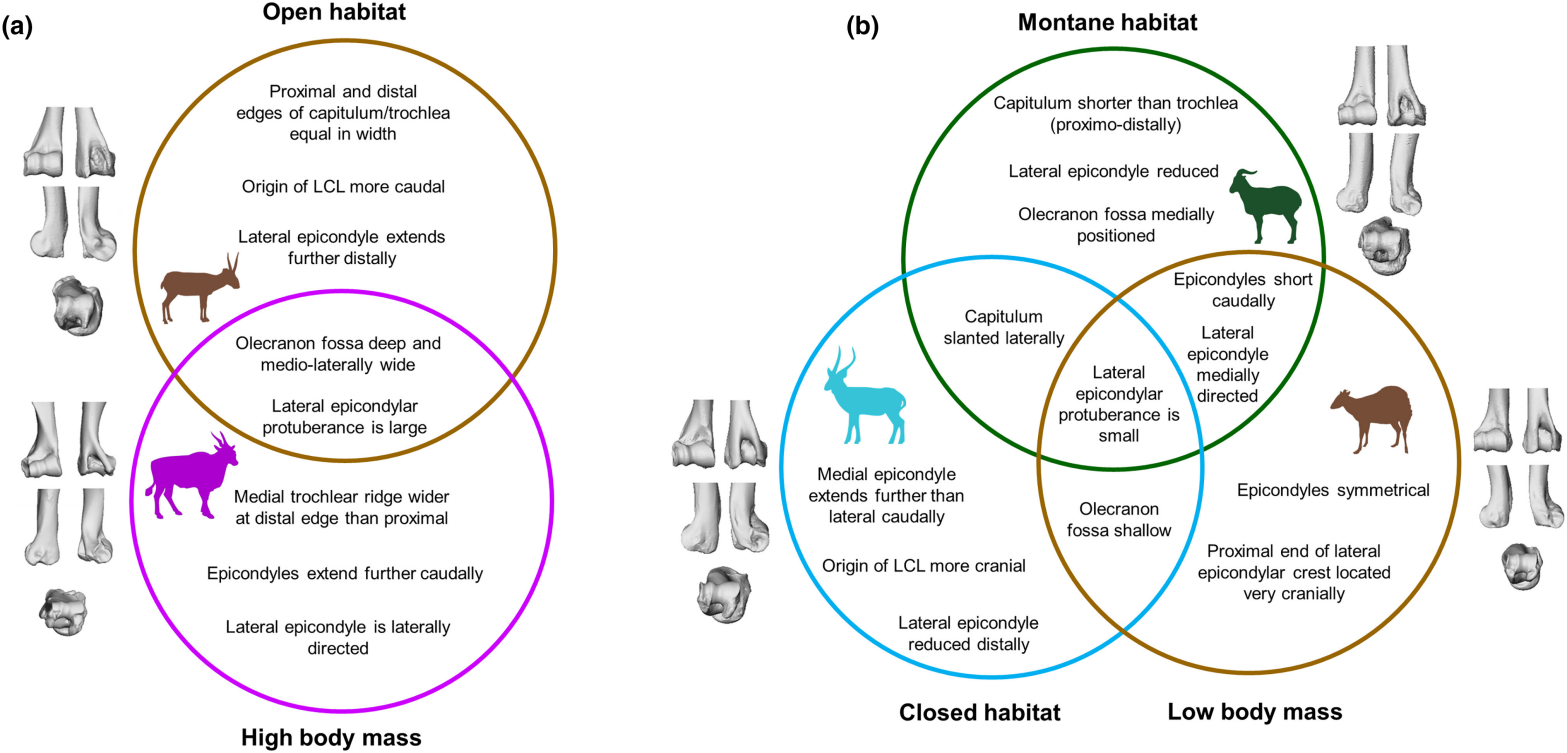
Notable distinguishing characteristics of extant bovid categories. Venn diagrams represent the overlaps in morphologies between categories in the analysis and features distinguishing the categories from one another. (a) The relationship between open‐living bovids and high body mass bovid morphologies; (b) the relationships between covered habitat‐preferring bovids, high body mass bovids and montane bovids. N.B. groups A and B are separated because there is no overlap between the two groups in relation to the features specified. Open habitat represented by *Saiga tatarica* (MNHN 195‐177); high body mass by *Taurotragus oryx* (NMNH 163308); montane by *Pseudois nayaur* (MNHN 1966‐136); covered habitats by *Kobus ellipsiprymnus* (NMNH 161989) and low body mass by *Cephalophus monticola* (AMNH M‐52752). Bovid silhouette sources as previously.

One important finding to note is that there are morphological similarities found between some habitat preference groups and some body mass categories. Particularly, there are shared characteristics between the bovids preferring the most open habitats and those with high body masses. Both open‐living bovids and those with high body mass experience high levels of stress on their long bones, and morphological similarities in the long bones of these two groups have been previously identified in extant bovids and linked to the strain resulting from this stress (Etienne et al., 2021). We find that, in these groups, the lateral epicondyle is enlarged (cranially and/or distally) with the caudal extremity laterally directed, the olecranon fossa is deep and medio‐laterally wide (particularly extending laterally), and the protuberance on the lateral epicondyle is enlarged in both groups. The lateral epicondyle provides the origin sites of almost all of the extensor muscles of the carpus and digits, so enlargement of the lateral epicondyle may reflect increased functional significance of these muscles. Equids possess specialised limb anatomy known as a ‘stay apparatus’ which allows them to stand for long periods while grazing and sleeping without any muscle activation (Dyce et al., 1996). Bovids are not known to possess this adaptation, so extensor muscles of the carpus and digits would be active constantly during grazing and standing in order to prevent the limb from collapsing. Open habitat grazers would, therefore, benefit from powerful extensor muscles, as would high body mass bovids whose extensors are resisting a large force when activated to keep the limb in extension. The depth of the olecranon fossa reflects increased stability of the joint, as it provides a deep articulation for the olecranon of the ulna. Though these features unite bovids preferring open habitats and those with high body mass, there are also several morphological differences between these groups which can distinguish them. Characteristics shared between groups, and characteristics which distinguish groups can be found in Figure 9. Notably, the bovids preferring open habitats and those with high body mass are largely distinguished from the other groups morphologically, and the main distinction is the presence/prominence of the lateral epicondylar protuberance in the former.

What is most clear from our results is that the distal humerus is a morphologically adaptable region in bovids, influenced by both an animal's phylogenetic affinity and its ecology. The results of this study provide evidence that habitat preference, body mass and tribe membership can be predicted to varying degrees of resolution from distal humerus morphology in extant bovids, with incidences of misclassification resulting in assignment to the next most similar group (e.g. GT misclassified as WBG, and 1–10 kg misclassified as 10–45 kg). This leads to confidence in using distal humerus morphology as a tool to better understand ecology in extinct bovids, such as *Rusingoryx*.

### 4.1 Rusingoryx atopocranion

*Rusingoryx*'s assignment to the tribe Alcelaphini and its humeral morphology indicating a preference for grassland/treeless habitats aligns with previous analyses of the Kenyan portion of the Lake Victoria basin which have reconstructed a flat, open plain dominated by grazers (particularly alcelaphines) during the Pleistocene (Faith et al., 2011; Pickford & Thomas, 1984; Tryon et al., 2014). We also support the results of an earlier ecomorphological study of the species which demonstrated many of its skeletal elements resembled those of extant grassland specialists (Kovarovic et al., 2021).

No formal estimate of body mass has been published for *Rusingoryx*, but based on the size of skeletal elements, it is putatively accepted to be of similar mass to modern wildebeest (Faith et al., 2011; Jenkins et al., 2017), and its mass has been speculatively estimated for quantitative purposes as 150 kg (Faith et al., 2012, 2013). We use the relationship between centroid size and body mass in extant bovids to estimate that *Rusingoryx* had a body mass of 125.89–141.25 kg, making it somewhat smaller than *C. taurinus* (blue wildebeest, 140–290 kg [Kingdon, 2013]), but well within the body mass range of closely related *C. gnou* (the white‐tailed gnu) which ranges from 110 to 180 kg depending on sex (Kingdon, 2013). If this estimate is reliable, the CVA of body mass correctly places the ‘mean *Rusingoryx*’ into the category 90–180 kg.

*Rusingoryx*'s distal humerus shares all of the identifying characteristics of open habitat bovids, and many of the characteristics of the high body mass, as well as the shared characteristics of open habitat and high body mass bovids, though the olecranon fossa in *Rusingoryx* is relatively shallow which is a characteristic of the covered habitat/low body mass bovids. The notable aspect in which *Rusingoryx* differs from high body mass bovids is an aspect in which it is more similar to low body mass bovids—the proximo‐medial and disto‐medial corners of the trochlea are vertically in line in cranial view, where the proximo‐medial corner is laterally positioned relative to the disto‐medial corner in heavy bovids. The most relevant characteristic is, of course, the lateral epicondylar protuberance, and the fact that this is large in *Rusingoryx* aligns it with the open habitat/high body mass bovids and distinguishes it from the covered habitat/low body mass/montane bovids. It is possible that in some way this enlargement of the lateral epicondylar protuberance beyond that seen in the extant bovids biomechanically compensates for features of *Rusingoryx* which are not aligned with open habitat preference or increased weight‐bearing, such as the relatively shallow olecranon fossa in *Rusingoryx*, overall providing high joint stability at the elbow.

Interestingly, an enlarged protuberance on the lateral epicondyle of the distal humerus has previously been identified to be related to strain at the elbow joint in bovids, but as a pathology. This pathology is known as ‘penning elbow’, and was first observed in archaeological sheep remains (though it has also been identified in other domestic bovids) where it has been hypothesised to be associated with incorrect husbandry practices such as keeping sheep in close proximity or on hard terrain (Baker & Brothwell, 1981; Bendrey, 2014; Clark, 2009; Clutton‐Brock et al., 1990; Upex & Dobney, 2012). In this condition, an osteophyte develops on the lateral epicondyle, usually in association with a corresponding osteophyte on the proximal radio‐ulna, and it is believed that this may develop in order to stabilise the joint which has become ‘mechanically compromised’ (O'Connor, 2008). This pathology is poorly understood, but the consensus has been that it appears in response to stress at the elbow joint, and this stress can be a result of life on hard terrain. As mentioned, *Rusingoryx* is believed to have lived on hard terrain, and evidence of its relatively small distal phalanges would imply greater joint stress in the lower limbs bearing an animal of its size. These could constitute mechanical stresses at the elbow joint which, though evolutionarily rather than pathologically, may have been mitigated by the enlarged epicondylar protuberance.

In the future, it would be greatly advantageous to investigate the biomechanical implications of the lateral epicondylar protuberance, perhaps using finite element analysis (FEA). This would allow testing of the hypothesis that the protuberance is related to stress mitigation at the elbow joint.

## 5 CONCLUSIONS

There is evidence of form‐function relationship between distal humerus morphology in extant bovids and habitat preference, body mass and tribe affiliation.

Extant bovids preferring very open habitats have a more robust distal humerus than those preferring cover or montane bovids. Bovids with high body mass also have a more robust distal humerus than those with low body mass. Shared morphologies between open habitat bovids and high body mass bovids are likely related to mitigating joint stresses.

Despite considerable individual variation, we find evidence to support *Rusingoryx*'s assignment to the tribe Alcelaphini, as well as evidence that it was best adapted for life in open grassland habitats based on distal humerus morphology. We also provide the first empirical estimate of *Rusingoryx* body mass at 123.4–140.4 kg, based on distal humerus size.

We posit that, in Rusingroyx, a large epicondylar protuberance (a feature most prominent in open‐living and high body mass extant bovids) is related to mitigating joint stress at the elbow due to the hardness of the terrain on which it lived, and *Rusingoryx*'s notably small distal phalanges for its size.

## AUTHOR CONTRIBUTIONS

KK and SCA contributed to the concept/design of the study. SCA, KK and WAB acquired the data. SCA analysed the data. SCA, KK and WAB interpreted the results. SCA and KK drafted the manuscript. SCA, KK and WAB critically revised the manuscript. SCA, KK and WAB approved the final article.

## Supporting information


Supplementary Material 1:

Supplementary Material 2:

Supplementary Material 3:

## Data Availability

The data availbility statement can now read: The data that support the findings of this study are openly available in Dryad at https://doi.org/10.5061/dryad.x69p8czsm.
