# Supplementary material for: A 3D geometric morphometric analysis of the bovid distal humerus, with special reference to Rusingoryx atopocranion (Pleistocene, Eastern Africa)
Source: J Anat. 2024 May 11;245(3):451–66. doi: 10.1111/joa.14062 (PMC11306763; doi:10.1111/joa.14062)
Supplement: Supplementary file 2 — Supplementary Material 2: [file JOA-245-451-s003.docx]

# Supplementary Material 2: Habitat and Body Mass Interaction


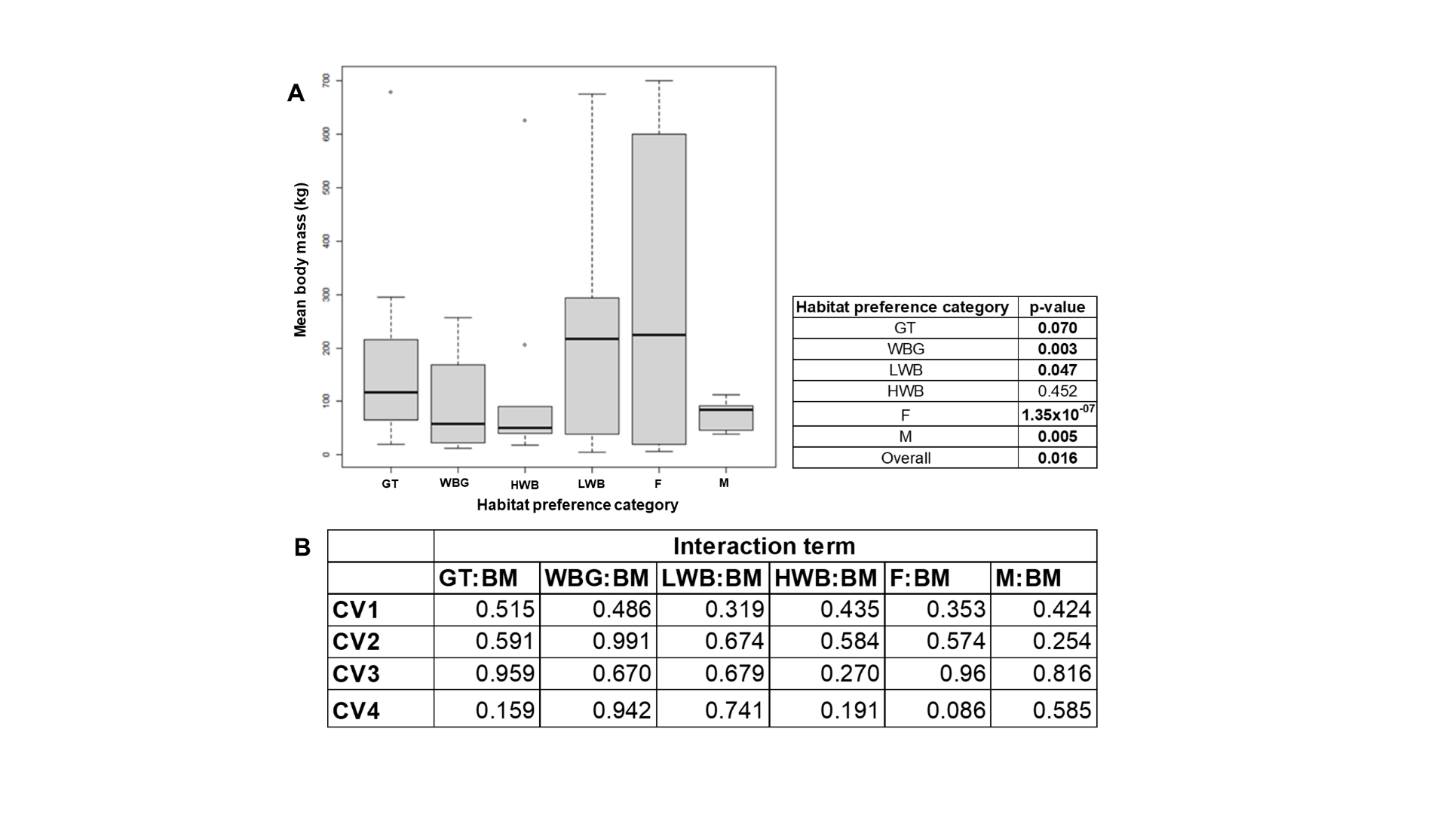


**Figure ESM1: Habitat preference and body mass interaction results**

A) Boxplot showing mean body mass for each habitat preference category, and table showing p-values for the significance of the relationship between body mass and habitat preference in each habitat preference category and overall.

B) Table showing the p-values resulting from an ANOVA investigating the effect of interaction between body mass and habitat preference on the first four axes of the habitat preference CVA.

N=55 (1 per extant species in dataset). Significant p-values are shown in in bold.

In this section, General Linear Models (GLMs) and Analysis of Variance (ANOVA) are used. A GLM regresses one or more independent variables against a dependent variable with the aim of modelling the predictive power of the independent variables. ANOVA generalises a t-test beyond two populations, and was used here to investigate the effect on the dependent variable of interaction terms between multiple independent variables.

It is important to consider that body mass and habitat preference may have an interaction, and that this may affect interpretation of morphological results. According to a General Linear Model (Fig. ESM1A), body mass (mean values in kg for each species) and habitat preference are significantly related overall (p = 0.016), with the forest category (F) being the most significantly related to body mass (p = 1.35x10-7, n = 7), followed by wooded bushland/grassland (WBG, p = 0.003, n = 13) and montane (M, p = 0.005, n = 9). Montane body masses appear to be the most constrained, with a mean of 72.4 kg and a standard deviation of 25.2 kg. Meanwhile, forest body masses are the most variable, with a mean of 283.0 kg and a standard deviation of 266.9kg, including the second smallest animal in the dataset (*Cephalophus monticola* at 6.3kg) and the second largest animal (*Bubalus bubalis* at 700kg).

However, the more important question is whether or not this relationship between body mass and habitat affects the previous morphology results. In order to assess this, another general linear model was produced for each of the first four CVs of the habitat preference CVA (as the independent variable) against habitat preference, body mass (mean values in kg for each species), and an interaction term for habitat preference and body mass (e.g. for CV1 the model can be represented in R as: CV1 ~ Habitat_preference + Body_mass + Habitat_preference*Body_mass). The interaction term significance results are shown in Fig. ESM1B). It can be seen that none of the interaction terms for habitat preference and body mass are significant on any of the first four CVs, indicating that the morphological changes identified in the analysis along each of these axes can be said to be related to habitat preference only and not affected by the interaction of body mass and habitat preference. An ANOVA was also performed on the habitat preference CVA data, and the interaction term was found not to be significant in any CV (CV1, p = 0.815; CV2, p = 0.692; CV3, p = 0.670; CV4, p = 0.255), indicating that the relationship between habitat preference and body mass does not play a significant role in the variance observed in the shape data.

In summary, there is a significant relationship between body mass and habitat preference in extant bovids, but this does not have a significant effect on the previously reported morphological results.
