# Supplementary material for: A 3D geometric morphometric analysis of the bovid distal humerus, with special reference to Rusingoryx atopocranion (Pleistocene, Eastern Africa)
Source: J Anat. 2024 May 11;245(3):451–66. doi: 10.1111/joa.14062 (PMC11306763; doi:10.1111/joa.14062)
Supplement: Supplementary file 3 — Supplementary Material 3: [file JOA-245-451-s001.docx]

# Supplementary Material 3: Allometric Shape Variation

In this section, linear and multivariate regressions are used. Linear regression is used to investigate the relationship between two linear variables, while multivariate regression is used to investigate the relationship between a multivariate variable and either another multivariate variable, or a linear variable.

Here we investigated allometric shape variation in the distal humerus of extant bovids. A multivariate regression of shape variation vs. centroid size (both outputs from MorphoJ, the former being a multivariate variable representing shape variation in the dataset on one axis) was performed to assess this. As can be seen in Figure ESM3A, there is evidence of a significant positive correlation (p < 0.001) between shape variation and centroid size; an indication that the heavier body mass categories have larger centroids and greater shape variation. Fig. ESM3B shows that shape variation generally increases with increasingly large body mass, seeming to plateau from the 360-575kg category.

In summary, these results indicate that the extant bovids with the larger distal humerus (which are also the heavier bovids), exhibit a large amount of size-related shape variation.


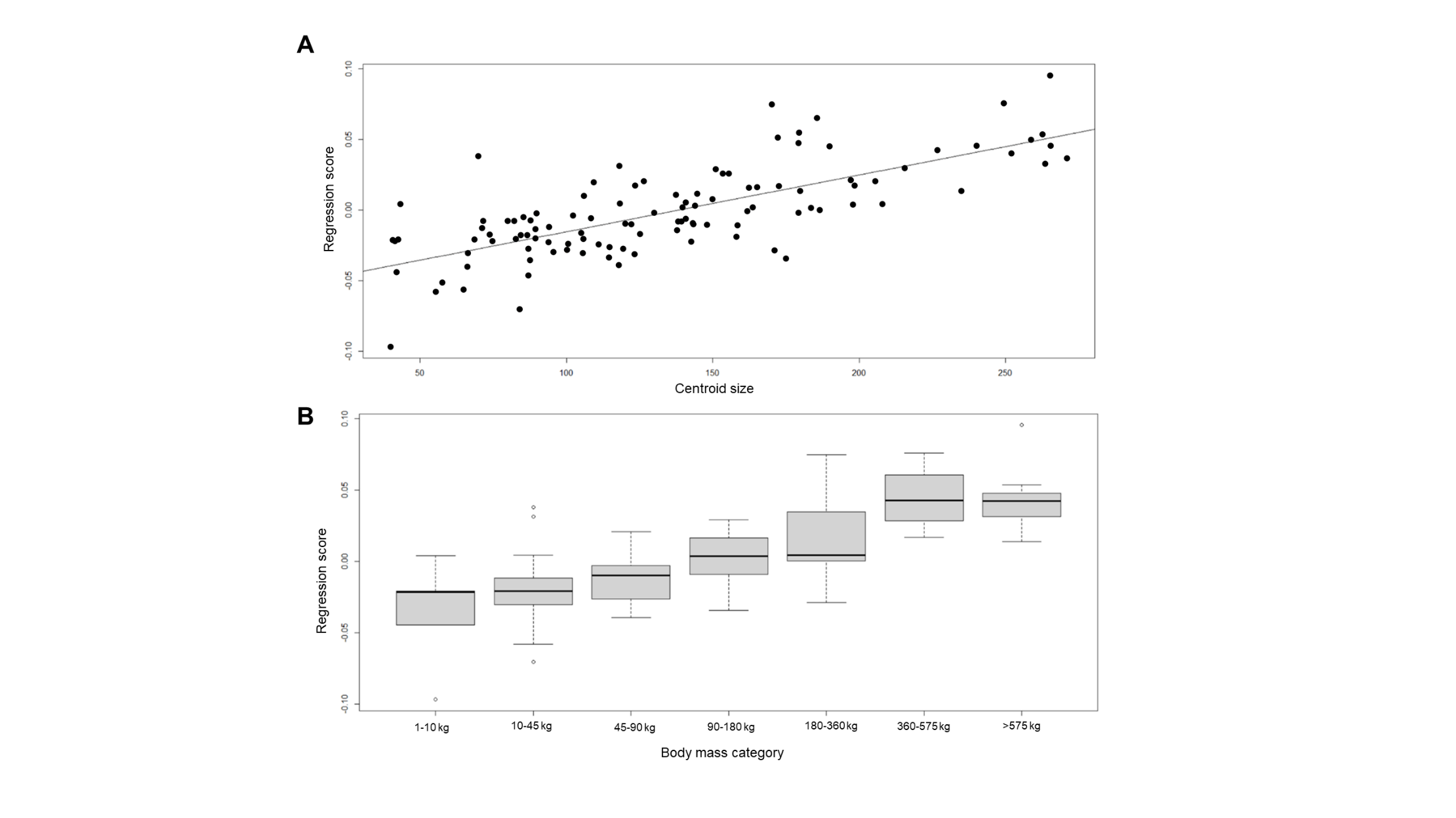


**Figure ESM3: Allometric scaling with distal humerus morphology in extant bovids**

A) Scatter plot of regression score against centroid size (following regression of Procrustes coordinates against centroid size). Shape variation = (4.023x10^-4^) x Centroid size + (-5.562x10^-2^), p < 0.01, adjusted r^2^ = 0.532, n=111

B) Boxplot of regression score in each body mass category (following regression of Procrustes coordinates against body mass category). p < 0.01
